# 

**Published:** 1895-10

**Authors:** 


### CONTENTS.

ORIGINAL ARTICLES:
- The Therapeutics of Colic. By Dr. W. L. Williams . . . . . . . 613
- Points in Practice. By Junius H. Wattles, D.V.S. . . . . . . . 628
- Plural Pregnancy, Involving a Question as to Sire. By Dr. W. H. Harbaugh . 632
- An Improved Method of Plantar Neurotomy. By M. H. McKillip, M.D., V.S., 638
- Physical Diagnosis. By Jacob Helmer, D.V.S. . . . . . . . . 640
- A Suggestion or Aid to the Eradication of Tuberculosis by a Practical and Profitable Method. By A. S. Heath, M.D., V.S. . . . . . . . . 646
- Paracentesis Abdominalis. By James A. Waugh, V.S. . . . . . . 649

SELECTIONS:
- Glanders and Mallein. By H. D. Gill, V.S. . . . . . . . . . . 653
- An Improved Apparatus for the Administration of Chloroform to Horses, with Remarks Thereon . . . . . . . . . . . . . . . 656
- On the Employment of the Horse-Chestnut in Pulmonary Emphysema in the Horse. By M. H. Benjamin . . . . . . . . . . . . 663

New York State Board of Veterinary Examiners . . . . . . . . . . 667

REPORTS OF CASES:
- Treatment of a Tumor with Iodic Acid. By Dr. Wilfried Lellman . . 668
- An Interesting Case. By William J. Waugh, V.S. . . . . . . . 670
- Hypertrophy of the Clitoris. By A. W. Clement, V.S. . . . . . . 671

Commencement Exercises, Veterinary Department, Detroit College of Medicine . . 672

CONTROL WORK:
- Tuberculosis—Anthrax—Uniform Movement for Dealing with Tuberculosis . 672–674

Echoes of the Convention . . . . . . . . . . . . . . 675

A Protestation. By A. Liautard, M.D., V.M. . . . . . . . . . . 677

EDITORIALS:
- Facts, Not Forebodings—U. S. V. M. A., 1895—New York's Board of Examiners—McKillip Veterinary College—Association of Veterinary Faculties—Our Journey Westward . . . . . . . . . . . . . . 679–688

PERSONALS . . . . . . . . . . . . . . . . . 689

SOCIETY MEETINGS AND PROCEEDINGS:
- Indiana Association of Veterinary Graduates—Pennsylvania State Veterinary Medical Association—Keystone Veterinary Medical Association . . . . 690–696

